# Automatic Tube Compensation versus Pressure Support Ventilation and Extubation Outcome in Children: A Randomized Controlled Study

**DOI:** 10.1155/2013/871376

**Published:** 2013-02-26

**Authors:** Ahmed Saad El-din El-beleidy, Asser Abd EL-Hamied Khattab, Seham Awad El-Sherbini, Hebatalla Fadel Al-gebaly

**Affiliations:** ^1^Department of Pediatrics, Faculty of Medicine, Cairo University, Cairo, Egypt; ^2^Specialized New Children Hospital, Faculty of Medicine, Cairo University, Ali Basha Ebrahim St., P.O. Box 11562, Cairo, Egypt

## Abstract

*Background*. Automatic tube compensation (ATC) has been developed to overcome the imposed work of breathing due to artificial airways during spontaneous breathing trials (SBTs). *Objectives*. This study aimed to assess extubation outcome after an SBT (spontaneous breathing trial) with ATC compared with pressure support ventilation (PSV) and to determine the risk factors for extubation failure. *Methods*. Patients ready for extubation were randomly assigned to two-hour spontaneous breathing trial with either ATC or pressure support ventilation. *Results*. In the ATC group (*n* = 17), 11 (65%) patients passed the SBT with subsequent extubation failure (9%). While in PSV group (*n* = 19), 10 (53%) patients passed the SBT with subsequent extubation failure (10%). This represented a positive predictive value for ATC of 91% and PSV of 90% (*P* = 0.52). Five (83%) of the patients who failed the SBT in ATC group were reintubated. This represented a higher negative predictive value for ATC of 83% than for PSV which was 56%. None of the assessed risk factors were independently associated with extubation failure including failed trial. *Conclusion*. ATC was equivalent to PSV in predicting patients with successful extubation. A trial failure in ATC group is associated with but does not definitely predict extubation failure.

## 1. Introduction

 Prolonged and unnecessary delay in tracheal extubation result in increased complication rates for patients receiving mechanical ventilation including airway trauma, chronic lung disease, ventilator associated pneumonia, and increased hospital costs [1]. On the other hand-premature discontinuation carries a set of problems involving difficulty in establishing airways and compromised blood gas exchange [2].

Different methods, including clinical trials and calculated indices, have been developed to evaluate patients on mechanical ventilation and predict the optimum time to make the weaning decision [3]. These methods include, tolerances of spontaneous breathing trials (SBTs), counting the respiratory rate, observation of work of breathing, and many other calculated indices such as the oxygenation index, measurement of the tidal volume and dynamic compliance, and the commonly used rapid shallow breathing index. However, some of these indices may be misleading, cost-effective, and requiring highly sophisticated equipments [4]. 

 Recently, a tolerance of a spontaneous breathing trial while the patient receives varying levels of ventilatory support including continuous positive airway pressure (CPAP), low-level pressure support ventilation (PSV), or very recently automatic tube compensation (ATC) is a new clinical test that has been considered an evidence-based strategy to predict successful weaning from assisted ventilation [5].

 The level of support may be relevant to whether the breathing trial is tolerated, because it has been argued that, for some patients, weaning failure may be attributable to the respiratory load imposed by the endotracheal tube [6]. Automatic tube compensation (ATC) is a recent weaning mode of mechanical ventilation that has been developed to overcome the imposed work of breathing due to artificial airways. It delivers the exact amount of resistive load of the endotracheal tube for the flow measured at time, without affecting the patient's breathing pattern [7]. It potentially simulates spontaneous breathing without endotracheal tube, so it has been designated as “electronic extubation”. This mode of ventilation thus seems ideally suited for use during the weaning period [8].

 PSV has been also widely used in the performance of a spontaneous breathing trial and has been shown to compensate for the additional work of breathing imposed by the endotracheal tube [9]. However, some studies have shown that compared with PSV, ATC was more effective in overcoming the work of breathing necessary to overcome endotracheal resistance and resulted in more significant predictive values for successful weaning and extubation [10].

Pediatric and adult studies evaluating the efficacy of SBTs have not systematically extubated patients who failed the breathing trial. Therefore, the ability of a failed SBT to predict the need for ventilator support was not formally assessed, except in a previous study by Chavez et al. in pediatric population [11]. In our study, we assessed the sensitivity of both ATC and PSV in predicting extubation outcome, and we also assessed the outcome of failed SBTs.

## 2. Patients and Method

### 2.1. Population and Setting

The study was conducted in Cairo University Pediatric Hospital; pediatric intensive care unit, 9-bed capacity. The study period extended from May 2011 to February 2012. 

 In this prospective, randomized, controlled study, we screened 47 mechanically ventilated patients. 

#### 2.1.1. Inclusion Criteria

 Patients were eligible for enrolment in the study if they met the following criteria judged by the intensive care doctors: (1) required mechanical ventilation for more than 24 hours; (2) fulfilling weaning criteria, which was defined in our PICU as follows: low ventilator rate [6–8] or less; fraction of inspired oxygen (FIO_2_) ≤ 40; level of positive end expiratory pressure (PEEP) [3, 4]; Improvement of the cause of respiratory failure; oxygenation index (OI) ([mean air way pressure × FIO_2_]/PaO_2_) < 5; the need for bronchial suction is ≤2 for the last 8 hours; with stable vital, neurological and metabolic status. 

#### 2.1.2. Exclusion Criteria

 Included the following: (1) duration of mechanical ventilation before enrolment is 24 hours or less; (2) patients receiving high doses of sedations or vasoactive drugs; (3) patients with disturbed conscious level despite improvement of lung pathology; (4) patients who developed laryngeal edema after extubation; (5) patients with pulse oxygen saturation < 90%, PH < 7.3 and PaCO_2_ > 50 mm Hg during the trial.

Full history and data analysis including sex, age, weight, cause of mechanical ventilation, period of mechanical ventilation, length of stay in the PICU, bronchodilators, pediatric risk of mortality score (PRISM III) [12] on day 1 admission, blood gases, pretrial oxygenation index, and ventilator setting parameters including: level of positive end expiratory pressure (PEEP); pretrial ventilator rate; pretrial FIO_2_ were recorded. 

### 2.2. Study Protocol and Weaning Procedures

Patients screened were randomly assigned in a blinded fashion with the use of opaque, sealed envelopes, to undergo two-hours spontaneous breathing trial with ATC (patients breathed through the ventilator circuit using continuous positive airway pressure of 5 cmH_2_O, FIO_2_ less than 0.5 with the addition of ATC 100%; the ATC group) or PSV (patients breathed through the ventilator circuit using flow triggering and continuous positive airway pressure of 5 cmH_2_O, FIO_2_ less than 0.5, PS adjusted for endotracheal tube size (ETT) (ETT size 3.0–3.5 = PS of 10 cmH_2_O; ETT size 4.0–4.5 = PS of 8 cmH_2_O; ETT size ≥ 5.0 = PS of 6 cmH_2_O) [13]; the PSV group). These parameters were maintained throughout the trial. The spontaneous breathing trial was performed using the Puritan-Bennett 840 ventilator which compensate automatically for air leaks (we do not use cuffed ETTs) and was newly introduced in our PICU; previously used ventilator was Newport E150. The SBT was conducted by a respiratory therapist and nurse in the absence of the attending or other intensive care staff.

Physical signs including heart rate; respiratory rate; mean arterial blood pressure; spontaneous expiratory tidal volume (mL/kg/sec); evidence of work of breathing; increased frequency of suction; pulse oxygen saturation blood gases were recorded during the trial. 

Features of poor tolerance and weaning failure included respiratory rate outside the acceptable range for their age [14]; increase in heart rate of more than 20% with respect to baseline on mechanical ventilation; increase or decrease in mean blood pressure of more than 20% of baseline; signs of increased respiratory work (i.e., retractions, use of accessory respiratory muscles, paradoxical breathing); pulse oxygen saturation < 90% and/or PH < 7.3 and/ or PCO_2_ > 50 mm Hg. When one of these findings occurred during the trial, the respiratory therapist terminated the trial to the previous ventilator settings. For patients with metabolic or respiratory acidosis during the trial, weaning criteria was revised with ICU doctors, and these patients were excluded from the study. 

Patients who passed the 2 hr trial were extubated by the respiratory therapist, and patients who failed the trial and were included in our study were recorded to be extubated within the next 24 hr by the intensive care doctors who were blinded to the results of the trial and the study aims.

Weaning was considered successful if reintubation was not required within 48 hr of extubation (successful extubation group). Failure to wean was defined as reintubation within 48 hr of extubation (extubation failure group).

### 2.3. Ethics

Informed consent was obtained from at least one parent or legal guardian for each patient before enrollment. The study design conformed to the Revised Helsinki Declaration of Bioethics [15] and was approved by the Scientific Ethics Committee of Department of Pediatrics, Faculty of Medicine of Cairo University.

### 2.4. Statistical Analysis

Data was analyzed using Statistical Package for Special Science software computer program version 16.0 (SPSS Inc., Chicago, Illinois, USA). Continuous variables were expressed as median, minimum, and maximum. Categorical variables were expressed as number (*n*), percent (%) and were compared using the chi-square test or Fisher's exact test, as indicated. Continuous variables were compared using Mann-Whitney test and Kruskal-Wallis ANOVA, as indicated. Multivariate regression analysis was used to test the association between multiple quantitative and qualitative independent variables with the dependent variable. *P* value less than or equal 0.05 was considered statistically significant.

## 3. Results

 Out of 47 screened patients, only 36 were enrolled in the study, 6 patients were excluded as they were reintubated due to laryngeal edema, and in the other 5 trial was terminated due to metabolic or respiratory acidosis during the trial. Of the 36 patients enrolled in the study, 17 were weaned from mechanical ventilation on ATC (ATC group) and 19 were weaned on PSV (PSV group).

Admission diagnosis of patients enrolled was as follows: lower respiratory tract infections (*n* = 9); interstitial lung disease (*n* = 3); postoperative (*n* = 5); status epileptics (*n* = 2); encephalitis (*n* = 2); after arrest (*n* = 1); history of poison intake (*n* = 2); autoimmune diseases (*n* = 2); Guillain-Barré syndrome (*n* = 2); endocrinal disorder (*n* = 1); septic shock (*n* = 3); gastroenteritis and shock (*n* = 2); myocarditis (*n* = 1); immunodeficiency (*n* = 1). 

Baseline characteristics are shown in Table 1. Respiratory and hemodynamic characteristics during the spontaneous breathing trial are shown in Table 2. There were no significant differences between the ATC and PSV groups in any of the compared items. 

**Figure 1:**
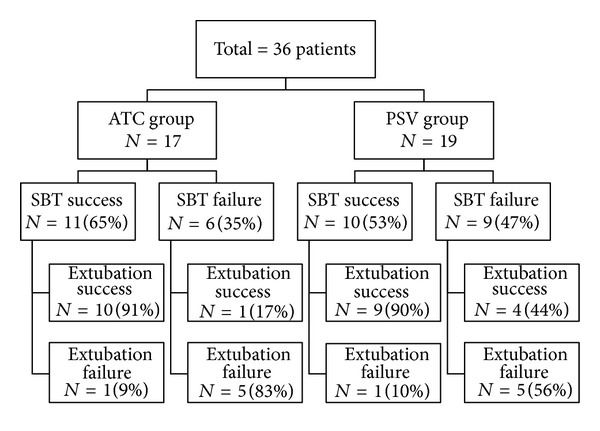
Extubation outcome in the two groups: Automatic tube compensation (ATC) versus pressure support ventilation (PSV).

The course and outcome of the study population are summarized in Figure 1. In the ATC group 11 of 17 (65%) passed the SBT compared with 10 of 19 (53%) in the PSV group, but this difference was not statistically significant, (*P* = 0.69). Out of 36 patients 12 (33.3%) were reintubated within the first 48 hours after extubation. Failed extubation was equal in both groups (*P* = 0.9). Causes of reintubation were hypoxemia (*n* = 5), disturbed conscious level (*n* = 3), and new sepsis and pneumonia (*n* = 4). There were no significant difference in the causes of reintubation between the two groups (*P* = 0.46). Mean length of stay in ATC group was 20.9 ± 14.4, while in PSV group was 21.35 ± 11.8 with no significant difference (*P* = 0.78).

Our study showed that successful completion of the SBT had a greater predictive value for successful extubation than the predictive value of failed trial for extubation failure; successful completion of the SBT on ATC showed a 91%, sensitivity with a positive predictive value of 91% and specificity was 83% with a negative predictive value of 83% and accuracy 88%. While successful completion of the SBT on PS showed a 69% sensitivity for predicting successful extubation with a positive predictive value of 90% and specificity was 83% with a negative predictive value of 56% and accuracy 74% with no significant difference between the two groups (*P* = 0.52).

Table 3 shows the univariate analyses comparing patients who were successfully extubated and patients who failed extubation and reintubated there was a statistically significant association between reintubation and the following risk factors during the breathing trial: tachypnea, tachycardia, increased work of breathing, and failing ATC trial.

 By stepwise multivariate logistic regression analysis of significant risk factors among all the study group (No = 36), none of the estimated significant risk factors were independently associated with extubation failure. 

## 4. Discussion

In the current study, we compared extubation outcome using 100% ATC versus PSV during a spontaneous breathing trial for two hours. The baseline characteristics in both groups were similar. 

We found that 9 of 19 (47%) patients in the PSV group failed the breathing trial compared to only 6 of 17 (35%) patients in the ATC group. This observed difference of 12% between both groups however did not reach statistical significance (*P* = 0.69). The positive predictive values were nearly similar in both groups; 9 of 10 patients in the PSV (90%) passed the SBT and successfully extubated (maintained extubation for >48 hours) compared with 10 of 11 (91%) patients in the ATC group. These findings are similar to a study conducted by Cohen and his colleagues [6] comparing ATC with PSV during a spontaneous breathing trial in adults. They found that patients who failed SBT in PSV group were higher than those in ATC group; however the difference was not significant. They found that PSV had a higher PPV predicting patients with successful extubation than ATC (PSV, 85% versus 80%); however, the difference was not significant (*P* = 0.87). 

In another study comparing ATC with PSV, the author found no significant difference in extubation outcome between the two groups; however, he did find that half of the patients who failed a breathing trial with PSV tolerated a subsequent trial with ATC and were successfully extubated [7]. These findings can be explained by the fact that ATC may provide more complete support. This is supported by a previous study in which the authors assessed the accuracy of the compensation provided by PSV and ATC relative to the endotracheal tube-related pressure dissipation. They found that the difference between the theoretical pressure required to overcome the endotracheal tube resistive properties and the actual pressure delivered by the ventilator was lower and negligible when ATC was applied during a spontaneous breathing trial when compared with PSV [10].

In our study, we found that rate of patients who failed the SBT in ATC group and reintubated was higher than that of PSV group. The negative predictive values for successful extubation were 83% for ATC versus 55% for PSV; however, this difference was not statistically significant (*P* = 0.52). This may be attributed to small number of cases in each group in addition to the nearly similar accuracy of the two groups (74% for PSV and 88% for ATC). The reason for this low negative predictive value in PSV group may be secondary to mechanical factors such as endotracheal tube discomfort, increased work of breathing caused by the augmented resistive force imposed by a small endotracheal tube, and inability to overcome this load due to in part the use of a relative low-level pressure in the PSV group used in this study. Pressure support level for each ETT size used in this study defined by Randolph et al. [13] may need to be reevaluated in another study with a large population size. These findings were similar to a study conducted in pediatric patients by Chavez et al. [11], who found that failed SBTs, using a flow-inflating bag to provide a low constant pressure of 5 mmHg, did not accurately predict extubation failure; however, this study did not compare the outcome of different pressure supported breathing trials. 

The reintubation rate for the whole studied patient was 33.3% which was higher than recent suggestions, where extubation rate of 15 to 29% implies an acceptable balance between performing premature extubation and unsuccessfully prolonged mechanical ventilation [16–18]. The finding that a significant number of patients who successfully passed the SBTs and extubated subsequently required reintubation merits further considerations. Patients with higher severity scores of illness at admission and those with higher incidence of nosocomial infections are at increased risk of extubation failure. Our relatively high incidence of reintubation rate could be in part due to the high incidence of nosocomial pneumonia and the development of new sepsis among patients under study, a finding which is consistent with other similar studies [19–22]. 

A potential limitation to our study was the small number of the study population which was related to the low turnover rate due to prolonged length of stay of our patients; median length of stay in the PICU in PSV group was 19 days (4–52 days), and median length of stay in the PICU in ATC group was 20 days (4–50 days).

Finally, the use of either ATC or PSV for prediction of extubation outcome in general ICU populations was, reliable and did not require special monitoring or complex data collections. Both have accepted positive and negative predictive values for successful extubation. 

## Figures and Tables

**Table 1:** Baseline characteristics of patients in each group before the spontaneous breathing trial.

	PS (19)	ATC (17)	*P* value
Sex:			
Female: number (%)	6 (31.6%)	7 (41.2%)	0.549
Male: number (%)	13 (68.4%)	10 (58.8%)	
Age (yrs): median (range)	0.83 (0.08–13)	1.25 (0.33–8)	0.068
Cause of mechanical ventilation (MV):			
Acute respiratory failure: number (%)	12 (63.2%)	5 (29.4%)	
Neurological dysfunction: number (%)	3 (15.8%)	8 (47.1%)	0.08
Shock and sepsis: number (%)	4 (21.1%)	4 (23.5%)	
MV* duration before trial: median (range)	7 (2–40)	11 (3–30)	0.260
Pretrial rate: median (range)	10 (5–30)	8 (4–20)	0.150
Pretrial FIO_2_: mean ± SD*	40.53 ± 5.24	42.65 ± 6.15	0.272
Pretrial OI*: mean ± SD*	2 ± 1.05	2.24 ± 1.47	0.539
Bronchodilators: number (%)	10 (52.6%)	7 (41.2%)	0.492
PRISM III score* on day 1 admission: mean ± SD*	19.53 ± 10	20.24 ± 10.2	0.787

*MV: mechanical ventilation.

*SD: standard deviation.

*PRISM III score: pediatric risk of mortality score.

*OI: oxygenation index.

**Table 2:** Hemodynamic and respiratory parameters of patients in each group during 2 hr spontaneous breathing trial.

	PS (19)	ATC (17)	*P* value
Respiratory rate/min: median (range)	34 (22–62)	32 (20–60)	0.6
Tachypnea: number (%)	5 (26.3%)	4 (23.5%)	0.577
Spontaneous tidal volume (mL/kg/sec): median (range)	6.3 (2.3–12)	6.36 (1.2–9)	0.962
Heart rate: mean ± SD	145.26 ± 22.72	149.29 ± 23.09	0.601
Tachycardia: number (%)	8 (42.1%)	6 (35.3%)	0.470
Hypertension: number (%)	7 (36.8%)	4 (23.5%)	0.387
Hypotension: number (%)	1 (5.3%)	1 (5.9%)	0.729
Increase need for suction: number (%)	2 (10.5%)	2 (11.8%)	0.655
Increase work of breathing: number (%)	7 (36.8%)	6 (35.3%)	0.923
Pulse oxygen saturation (%): mean ± SD	97.84 ± 1.6	97.29 ± 2.14	0.373

**Table 3:** Risk factors for extubation failure during SBT in the study population.

	Failed	Successful	*P* value
Age (yrs): median (range)	1 (0.2–13)	0.87 (0.08–8)	0.213
Sex:			
Female	4 (30.8%)	9 (69.2%)	0.553
Male	8 (34.8%)	15 (65.2%)	
Tachypnea: number (%)	6 (66.7%)	3 (33.3%)	0.022
Tachycardia: number (%)	9 (64.3%)	5 (35.7%)	0.003
Hypertension: number (%)	5 (45.5%)	6 (54.5%)	0.259
Spontaneous tidal volume (mL/kg/sec): median (range)	5.45 (1.2–11.5)	6.35 (3.2–12)	0.298
Increased work of breathing: number (%)	8 (61.5%)	5 (38.5%)	0.010
Increase need for suction: number (%)	2 (50.0%)	2 (50.0%)	0.407
Bronchodilators: number (%)	4 (23.5%)	13 (76.5%)	0.238
Trial type: number (%)			
PS	6 (31.6%)	13 (68.4%)	0.813
ATC	6 (35.3%)	11 (64.7%)	
Failed PS trial: number (%)	5 (56%)	4 (44%)	0.259
Failed ATC trial: number (%)	5 (83%)	1 (17%)	0.03
Pretrial rate: median (range)	8 (6–19)	10 (4–30)	0.383
Pretrial FIO_2_: mean ± SD	43.75 ± 6.78	40.42 ± 4.87	0.099
Causes of M.V.:			
Acute respiratory failure	6 (35.3%)	11 (64.7%)	
Neurological dysfunction	4 (36.4%)	7 (63.6%)	0.850
Shock and sepsis	2 (25%)	6 (75%)	
Duration of M.V. before trial: median (range)	14.5 (3–33)	7.5 (2–40)	0.207
